# Familial dystonic Choreoathetosis associated with Biallelic *ATM* variants

**DOI:** 10.3389/fneur.2026.1973543

**Published:** 2026-10-27

**Authors:** Marek Godava, Lenka Satke, Pavel Otruba, Petr Kaňovský, Raymond L. Rosales, Václav Palata, Alena Šantavá

**Affiliations:** 1 Centre of Fetal Medicine, Genetics and Gynecology, FETMED s.r.o., Olomouc, Czechia; 2 Laboratory of Medical Genetics, SPADIA LAB a.s., Nový Jičín, Czechia; 3 Department of Neurology, University Hospital Olomouc & Faculty of Medicine and Dentistry, Palacký University Olomouc, Olomouc, Czechia; 4 Neuroscience and Research Center for Health Sciences, University of Santo Tomas, Manila, Philippines; 5 Department of Medical Genetics, University Hospital Olomouc & Faculty of Medicine and Dentistry, Palacký University Olomouc, Olomouc, Czechia

**Keywords:** *ATM*, choreoathetosis, dystonia, exome sequencing analysis, variant ataxia-telangiectasia

## Abstract

Combined dystonia represents a heterogeneous group of movement disorders with numerous genetic causes, making the diagnostic evaluation challenging. We report a 46-year-old woman with childhood-onset familial dystonic choreoathetosis who remained without a molecular diagnosis for almost four decades despite extensive neurological, metabolic, and genetic investigations. A similar movement disorder was present in her younger sister, suggesting a hereditary aetiology and autosomal recessive inheritance. The proband’s first symptoms appeared at 18 months of age as involuntary movements of the shoulders and hands, intermittent head jerks, and toe walking. The disorder progressed slowly during childhood and adulthood, with the subsequent development of cervical dystonia, generalized choreiform dyskinesias, gait impairment, truncal dystonia, and dysarthria, while cerebellar ataxia, telangiectasia, immunodeficiency, and respiratory involvement remained absent. Brain magnetic resonance imaging was repeatedly normal, and targeted genetic testing for common hereditary dystonias was negative. Initial clinical exome sequencing identified only a single pathogenic Ataxia-Telangiectasia Mutated gene (*ATM*) variant. Reanalysis of the exome several years later detected a second pathogenic *ATM* variant (NM_000051.4:c.8147 T > C), establishing the diagnosis of variant ataxia-telangiectasia caused by biallelic *ATM* variants. This case expands the phenotypic spectrum of variant ataxia-telangiectasia by demonstrating that childhood-onset dystonic choreoathetosis may precede the classical manifestations of the disease by several decades or occur in their absence. Furthermore, it highlights the diagnostic value of repeated reinterpretation of exome sequencing data in patients with unresolved hereditary movement disorders.

## 1 Introduction

Combined dystonia comprises a heterogeneous group of movement disorders in which dystonia is accompanied by additional hyperkinetic or hypokinetic features, including chorea, myoclonus, parkinsonism, or ataxia. Owing to considerable phenotypic and genetic heterogeneity, establishing the underlying diagnosis may be challenging, particularly in patients with childhood-onset disease and atypical clinical manifestations (1).

Biallelic pathogenic variants in the *ATM* gene cause ataxia-telangiectasia (AT), an autosomal recessive multisystem disorder classically characterized by progressive cerebellar ataxia, oculocutaneous telangiectasia, immunodeficiency, radiosensitivity, pulmonary complications, and an increased risk of malignancy. However, the clinical spectrum of *ATM*-related disease is broader than originally recognized. Patients with variant AT frequently exhibit milder neurological manifestations, slower disease progression, and atypical movement disorders that may precede or even overshadow the classical features of the disease (2–5).

Here, we report a woman with childhood-onset familial dystonic choreoathetosis who underwent an extensive diagnostic evaluation over several decades before repeated clinical exome analysis established the diagnosis of variant ataxia-telangiectasia. This case illustrates the phenotypic variability associated with *ATM* mutations and highlights the importance of periodic reanalysis of genomic data in patients with unresolved hereditary movement disorders.

## 2 Case presentation

The proband is a 46-year-old woman with a lifelong history of a slowly progressive hyperkinetic movement disorder. Her first symptoms appeared at approximately 18 months of age, when her parents noticed gentle pendulum-like involuntary movements of the shoulders and hands, intermittent head jerks, and toe walking. The abnormal movements disappeared during sleep and gradually improved by six years of age.

At the age of 10 years, she developed progressive stiffness of the hands, leading to impaired handwriting and drawing, with episodes consistent with writer’s cramp. During puberty, the involuntary movements recurred and gradually progressed, involving predominantly the lower limbs. She developed muscle stiffness, knee buckling, and in-toeing, which were more pronounced on the right side. Repeated neurological assessments resulted in a clinical diagnosis of familial dystonic choreoathetosis with myokymia. Trials of haloperidol and terguride were ineffective. Extensive metabolic investigations, including evaluation for Wilson disease, were unremarkable.

The family history was significant for a younger sister who developed a similar movement disorder characterized by cervical dystonia with involuntary “no-no” head movements. At nine years of age, the sister was diagnosed with T-cell acute lymphoblastic leukaemia and underwent chemotherapy and radiotherapy. She subsequently experienced three episodes of viral encephalitis, with encephalopathic changes demonstrated on brain MRI. She died at 19 years of age without undergoing genetic testing.

At 25 years of age, the patient was admitted for comprehensive neurological reassessment because of progressive deterioration of her movement disorder. She reported worsening involuntary movements of the head and limbs, increasing impairment of fine motor skills, and marked deterioration of handwriting, making routine daily activities difficult. The involuntary movements were consistently exacerbated by emotional stress. Neurological examination demonstrated cervical dystonia with left torticollis and mild retrocollis, hypertrophy of the left sternocleidomastoid muscle, generalized choreiform dyskinesias involving all four limbs, and a dystonic gait with in-toeing. Brain magnetic resonance imaging showed no structural abnormalities. Polymyographic examination demonstrated abnormal activity of the cervical muscles, allowing targeted treatment with EMG-guided botulinum toxin injections. This treatment resulted in sustained symptomatic improvement and was continued at approximately three-month intervals because of recurrent cervical dystonia.

At 29 years of age, botulinum toxin therapy was discontinued because of planned pregnancy. She subsequently delivered two healthy sons at 30 and 33 years of age. During this period, targeted molecular testing for *TOR1A* (exon 5 sequencing) and *SGCE* (sequencing of the whole coding sequence) was negative. Given the occurrence of a similar movement disorder in her sister, an autosomal recessive disorder was suspected; however, broader genetic testing was not routinely available at that time.

Following her second pregnancy, the disease gradually progressed. She developed increasing gait instability, frequent tripping, occasional falls, and worsening lower-limb dystonia with persistent knee buckling and in-toeing. Dysarthria gradually emerged and was consistently aggravated by emotional stress.

At the age of 35 years, she underwent a second comprehensive neurological evaluation. Examination demonstrated right torticollis with mild retrocollis, generalized choreiform dyskinesias without clinical evidence of cerebellar ataxia, and severe lingual dysarthria with markedly reduced maximum phonation time. Electromyography revealed chronic predominantly motor axonal polyneuropathy affecting the lower limbs, whereas repeat brain MRI remained normal. Regular botulinum toxin treatment was reinitiated with satisfactory symptomatic benefit. Clinical exome sequencing identified a single heterozygous pathogenic *ATM* variant (NM_000051.4:c.3802delG), but no second pathogenic variant was detected, precluding a definitive molecular diagnosis.

During the following years, truncal dystonia and generalized choreiform dyskinesias gradually progressed, leading to increasing impairment of gait and daily activities and eventually necessitating the use of a forearm crutch. During clinical assessment, an unusual feature of her speech disorder was observed: although dysarthria worsened under emotional stress, it consistently improved during prolonged continuous speech in a relaxed, non-stressful setting.

At 40 years of age, reanalysis of the clinical exome identified a second pathogenic *ATM* variant (NM_000051.4:c.8147 T > C), establishing the diagnosis of variant ataxia-telangiectasia caused by biallelic *ATM* pathogenic variants.

During subsequent follow-up, the disease course remained slowly progressive without the development of cerebellar ataxia, oculocutaneous telangiectasia, respiratory complications, visual or hearing impairment, or clinically significant immunodeficiency. Osteoporosis was diagnosed at 42 years of age. At the most recent follow-up, aged 46 years, she remained ambulatory with a forearm crutch and continued regular botulinum toxin treatment, which provided sustained symptomatic relief of cervical dystonia.

## 3 Discussion

This case illustrates an uncommon presentation of variant ataxia-telangiectasia (AT), in which childhood-onset combined dystonia remained the predominant neurological manifestation for more than four decades in the absence of cerebellar ataxia or other classical features of AT. The patient’s phenotype was characterised by slowly progressive dystonic choreoathetosis, cervical dystonia, gait impairment and dysarthria, whereas cerebellar signs, oculocutaneous telangiectasia, immunodeficiency, respiratory complications and visual impairment remained absent throughout follow-up. Such atypical presentations substantially broaden the phenotypic spectrum of *ATM*-related disease and represent a considerable diagnostic challenge (2–7).

Because cervical dystonia was the predominant manifestation for many years, the initial diagnostic work-up appropriately focused on the most common monogenic causes of isolated and combined dystonia. Molecular analysis of *TOR1A*, the most frequent cause of early-onset isolated dystonia, was performed first. More than 98% of patients with *TOR1A*-related dystonia carry the recurrent 3-bp deletion NM_000113.2:c.907_909delGAG p.(Glu303del), which accounts for 16–53% of early-onset dystonia in non-Ashkenazi populations (8). The patient tested negative for this pathogenic variant. Because her phenotype also included slowly progressive hyperkinetic movements suggestive of myoclonic dystonia, sequence analysis of *SGCE* was subsequently performed but did not identify any pathogenic variants. Extensive metabolic investigations, including evaluation for Wilson disease, were likewise unrevealing. The occurrence of a similar movement disorder in the patient’s sister strongly suggested autosomal recessive inheritance; however, in 2010, comprehensive molecular testing for autosomal recessive dystonia syndromes was not routinely available.

The introduction of clinical exome sequencing substantially broadened the diagnostic evaluation. Clinical exome sequencing, performed in 2016 (SSEL XT Focused Exome, Agilent) identified a single heterozygous pathogenic nonsense variant in *ATM*, NM_000051.4:c.3802del p.(Val1268Ter). As no second pathogenic variant was detected, these findings were considered insufficient to explain an autosomal recessive disorder. Five years later, exome reanalysis was performed using the Clinical Exome Solution from Sophia Genetics and their DDM software. This analysis confirmed the previously identified *ATM* variant and detected a second pathogenic missense variant, NM_000051.4:c.8147 T > C. Both variants were subsequently confirmed by Sanger sequencing, and cascade carrier testing was performed in the patient’s family.

To clarify why the second pathogenic variant had not been detected during the initial analysis, the original FASTAq files from 2016 were retrospectively reanalysed using the contemporary SeqOne Genomics platform. The updated analysis readily identified both pathogenic *ATM* variants, demonstrating that the second variant had been present in the original sequencing data but had not been recognised by the SureCall software (Agilent) analysis available at that time. Although the precise technical reason for this discrepancy remains unclear, this finding illustrates the continuous evolution of next-generation sequencing analysis and highlights the importance of periodic reanalysis of exome data in patients with unresolved hereditary disorders. Reanalysis of previously generated sequencing data has become increasingly valuable as bioinformatic pipelines, variant annotation, reference databases and ACMG interpretation criteria continue to evolve, leading to improved diagnostic yield without the need for repeat sequencing (2).

The identified genotype also provides valuable genotype–phenotype information. The c.3802del (p.Val1268Ter) variant is a well-established pathogenic loss-of-function allele predicted to undergo nonsense-mediated mRNA decay, resulting in near-absent *ATM* protein expression (9). In contrast, the missense variant c.8147 T > C has been associated with residual *ATM* kinase activity and milder neurological phenotypes consistent with variant AT (2, 3, 10). Previous studies have demonstrated that patients carrying this variant generally exhibit slower neurological progression, longer survival, fewer telangiectasias and lower frequencies of immunodeficiency and respiratory disease than patients with classical AT (6, 7). The relatively mild but slowly progressive neurological course observed in our patient is consistent with these genotype–phenotype correlations.

The family history further supports the diagnosis. The patient’s younger sister developed a remarkably similar movement disorder and subsequently died from T-cell acute lymphoblastic leukaemia during adolescence. Although molecular confirmation was not available, AT is associated with a markedly increased risk of haematological malignancies, particularly T-cell leukaemia and lymphoma (2, 6). It is therefore highly probable that she was affected by the same inherited disorder.

Despite sharing the same molecular diagnosis, considerable phenotypic variability has been reported among individuals with variant AT. Most published patients initially present with cerebellar ataxia, whereas dystonia is considerably less common as the predominant presenting manifestation (6). Furthermore, the combination of the c.3802del and c.8147 T > C variants has only rarely been reported (11). Compared with the previously published patient carrying the same genotype, our patient presented with childhood-onset familial dystonic choreoathetosis without cerebellar ataxia despite a similarly mild systemic phenotype. This observation further expands the recognised clinical spectrum associated with *ATM* mutations and illustrates the limited genotype–phenotype predictability in variant AT.

Several additional clinical observations deserve attention. The patient’s involuntary movements disappeared during sleep, a feature that has only rarely been described in patients with variant AT (12). Likewise, her dysarthria consistently improved during prolonged continuous speech in a relaxed environment despite marked deterioration under emotional stress. To our knowledge, this phenomenon has not previously been reported in variant AT and may represent an interesting observation for future studies of speech motor control and rehabilitation.

From a clinical perspective, this case emphasises that *ATM*-related disease should be considered in the differential diagnosis of childhood-onset combined dystonia, even in the absence of cerebellar ataxia, telangiectasia or immunodeficiency. Establishing the correct molecular diagnosis has implications extending beyond diagnostic certainty, including surveillance for malignancy, awareness of increased radiosensitivity, appropriate genetic counselling and family screening. Finally, this case demonstrates that negative or inconclusive exome sequencing should not be regarded as definitive. Periodic reinterpretation of existing genomic data using updated bioinformatic pipelines and current variant classification criteria may reveal previously undetected pathogenic variants and significantly improve the diagnostic yield of genomic testing.

## 4 Conclusion

This case expands the phenotypic spectrum of *ATM*-associated disease by demonstrating that variant ataxia-telangiectasia may present predominantly as childhood-onset familial dystonic choreoathetosis, even in the absence of cerebellar ataxia, telangiectasia, or immunodeficiency. Such atypical presentations may delay diagnosis for many years unless *ATM*-related disorders are considered in the differential diagnosis of combined dystonia.

Our findings also highlight the clinical value of repeated reanalysis of exome sequencing data in patients with unresolved hereditary movement disorders. As bioinformatic pipelines, variant databases, and interpretation strategies continue to evolve, previously undetected or unclassified pathogenic variants may become identifiable, ultimately establishing the correct molecular diagnosis.

Accurate genetic diagnosis is essential not only for confirming the underlying aetiology but also for guiding long-term clinical management, surveillance for malignancy, genetic counselling, and avoidance of unnecessary ionising radiation exposure in patients with variant ataxia-telangiectasia.

## Data Availability

The raw data supporting the conclusions of this article will be made available by the authors, without undue reservation.
